# Guidelines for outsourcing collective catering services in the Friuli Venezia Giulia region: Giulio Barocco

**DOI:** 10.1093/eurpub/ckac131.227

**Published:** 2022-10-25

**Authors:** G Barocco, M Palei, E Croci, A Savoia, M Mauro, F Stuto

**Affiliations:** 1 Servizio Igiene Alimenti e Nutrizione, Azienda Sanitaria Universitaria Giuliano Ison, Trieste, Italy; 2 Servizio Prevenzione, Sicurezza Alimentare, Direzione Centrale Salute, Regione FVG, Trieste, Italy; 3 Servizio Igiene Alimenti e Nutrizione, Azienda Sanitaria Universitaria Friuli Centra, Udine, Italy; 4 Servizio Igiene Alimenti e Nutrizione, Azienda Sanitaria Friuli Occidentale, Pordenone, Italy

## Abstract

**Issue:**

Many stakeholders involved in collective catering services (CCS) of schools, hospitals, elderly care homes, and workplaces of the autonomous region of Friuli Venezia Giulia (ARFVG) have reported some non-compliance with the nutritional, environmental or social standards required by public administrations (PA) procurement contracts (PC), which included the recommendations of the WHO, EU, Ministry of Health, and Ministry of the Environment (MHE).

**Description of the problem:**

The ARFVG prevention plans have set the goal to overcome the critical issues of the regional CCS which serve over 10% of the population (130,000 meals a day). Between 2019 and 2021, a multidisciplinary group of experts from health services and from the association of 215 regional municipalities developed the guidelines (GL) for outsourcing of CCS. The aim was to support PA in the drafting of PC, evaluation of offers and verification of contractual performance. Reference was made to Dir. 2014/24/EU, EU green public procurement criteria for food, catering services and vending machines, the national lines for CCS of MHE. The GL are characterized by the integration between the tender document models and the 8 qualification areas detailed in: restructuring; supplies; environmental impact and sustainability; production processes; personnel qualification and training; nutritional standards; customer satisfaction; and verification and research of quality.

**Results:**

Two regional central purchasing bodies have already used the GL to draft the PC for school CCS of 26 municipalities and for the whole hospital CCS of RAFVG. The application of guidelines in PC has introduced homogeneous high level qualification and verification standards for the CCS of the RAFVG.

**Lessons:**

To ensure the application of food, nutritional, environmental and social policies in CCS, it is essential to activate networks between professionals from different sectors in order to share tools to achieve the common sustainable development goals.

**Key messages:**

• To integrate food, nutritional, environmental and social regional policies into collective catering services, it is essential to apply systemic tools shared by all Public Administrations.

• Central purchasing bodies have the potential to introduce homogeneous and shared high level qualification and verification standards for collective catering services.

